# Influence of a home-based exercise program on the urine pH in elderly female subjects: a secondary analysis of a randomized controlled trial

**DOI:** 10.1186/s11556-017-0176-4

**Published:** 2017-05-16

**Authors:** Yuichiro Nishida, Keitaro Tanaka, Megumi Hara, Noriko Hirao, Hiroaki Tanaka, Takuro Tobina, Masaharu Ikeda, Hiroshi Yamato, Masanori Ohta

**Affiliations:** 1 Department of Preventive Medicine, Faculty of Medicine, Saga University, Nabeshima 5-1-1, Saga, 849-8501 Japan; 2 Laboratory of Exercise Physiology, Faculty of Sports and Health Science, Fukuoka University, Fukuoka, Japan; 3 Faculty of Nursing and Nutrition, University of Nagasaki, Nagasaki, Japan; 4 Fukuseikai Minami Hospital, Fukuoka, Japan; 5 Department of Health Development, Institute of Industrial Ecological Science, University of Occupational and Environmental Health, Kitakyushu, Japan; 6 Department of Food and Health Sciences, International College of Arts and Sciences, Fukuoka Women’s University, Fukuoka, Japan

**Keywords:** Older females, Uric acidification, Aerobic exercise

## Abstract

**Background:**

A low urine pH is a characteristic metabolic feature of metabolic syndrome and type 2 diabetes. The purpose of the current study was to investigate the effects of a 12-week home-based bench step exercise on the urine pH status of elderly female subjects.

**Methods:**

The current study is a secondary analysis of a randomized controlled trial (RCT) in which 59 postmenopausal female subjects were randomized to either the exercise group (*n* = 29) or the control group (*n* = 30). The subjects in the exercise group were instructed to perform home-based exercises using a bench step at the anaerobic threshold (AT), with a goal of performing ≥140 min/week at home for 12 weeks. The subjects in the control group were instructed to not change their normal lifestyle. Urine was collected after overnight fasting, and the urine pH was measured using a urinary test strip. The inter-group-differences at baseline and the pre-post changes within groups were assessed using the Mann-Whitney *U* test and Wilcoxon’s signed-rank test, respectively. Additionally, the difference in the post-intervention urine pH levels of the two groups, adjusted for the pre-intervention values (the estimated effect size) and the precision (95% confidence intervals) were investigated using an analysis of covariance.

**Results:**

The pre-post comparison of the urine pH data using Wilcoxon’s signed-rank test showed a significant increase in the urine pH levels of the exercise group (*p* < 0.05); there was no significant change in the urine pH levels of the control group. However, the estimated effect size (0.15) was small and the confidence interval straddled 0 (−0.25–0.55).

**Conclusions:**

Based on the results of the current secondary analysis of an RCT, we could not clearly conclude that exercise has a beneficial effect on the urine pH. Further well-designed RCTs should be conducted to determine whether aerobic exercise is truly able to ameliorate urine acidification.

**Trial registration:**

The study was retrospectively registered in the University Hospital Medical Information Network (UMIN) as “Effect of step exercise on aerobic fitness and progression of atherosclerosis in the elderly” under the registration number UMIN 000026743 (the date of registration: March 28, 2017).

## Background

People with type 2 diabetes and metabolic syndrome have been reported to have acidic urine [1–3]. An increase in the number of metabolic syndrome abnormalities is associated with an increase in the degree of urine acidification [3]. A lower urine pH is also correlated with a higher body mass index (BMI), triglyceride (TG), serum uric acid, and insulin resistance index values [3, 4]. These studies used a low urine pH as a sign of metabolic acidosis and indicated that acidic urine is a characteristic feature of the common metabolic diseases [1–5]. Furthermore, it has been known for a long time that even in healthy humans, very mild degree of chronic metabolic acidosis is sufficient to induce insulin resistance [6, 7].

A low body fluid pH or metabolic acidosis is considered to be a crucial factor in the induction of metabolic disorders, such as insulin resistance and type 2 diabetes [8, 9]. It is generally possible that the acidification of body fluid is unfavorably involved in the mechanisms of metabolic diseases, since a low pH can cause a number of abnormalities in various fundamental cell functions, such as abnormally low enzymatic activity and impaired protein-protein interaction [8]. For instance, a lower extracellular pH levels are associated with lower levels binding between insulin and insulin receptors, a lower level of insulin receptor activation (or phosphorylation), and the decreased activation of other key proteins that are involved in the muscle insulin signaling pathway [8, 10]. It should be noted that, even in healthy subjects, greater body fluid acidity has been associated with an increased risk of developing type 2 diabetes [11]. Thus, maintaining a proper pH status is considered to be crucially important for preventing metabolic diseases [8, 9].

In peripheral metabolic tissues such as skeletal muscle, the main proton sources are lactic acid (lactate^–^/H^+^), pyruvic acid (pyruvate^–^/H^+^), and beta-hydroxybutyric acid (beta-hydroxybutyrate^–^/H^+^) [8, 9]. The pH of body fluids (e.g., blood and urine) is determined by the amount of protons (H^+^) produced in the metabolic tissues, and the elevated production of the abovementioned organic acids–as sources of protons–is considered to be partly caused by an impaired mitochondrial function [8, 9]. The protons that are dissociated from organic acids are mainly buffered by bicarbonate ions (HCO_3_^-^) within cells, but the protons that are not eliminated are extruded to the extracellular fluid which has little buffering capacity, and extracellular fluid protons will be transported to the circulating blood, which contains abundant protein buffers (albumin and hemoglobin [Hb]). If these circulating protons cannot be removed by the blood buffers, they will ultimately be excreted from the body via urine and expiration. It is therefore conceivable that urine acidification is originally attributed to the overproduction of organic acids in the peripheral metabolic tissues such as skeletal muscle, which presumably occurs due to the impairment of the mitochondrial function [8, 9].

The lower expression of mitochondrial genes in the skeletal muscle has been reported in people with insulin resistance and type 2 diabetes [12], whereas moderate exercise at the anaerobic threshold (AT) intensity has been shown to induce a number of muscle genes that are involved in the mitochondrial functions in healthy adults [13]. A moderate home-based step exercise program is reported to be a safe, effective, and practical exercise regimen for improving aerobic fitness in elderly individuals [14]. Additionally, we recently showed that moderate step exercises at the AT in combination with the administration of branched-chain amino acids not only enhances the aerobic capacity but may also improve the glycemic control in elderly patients with liver cirrhosis [15].

In the current study, the main outcome measure was the urine pH. The secondary outcome measures included several metabolic parameters (TG, glucose, insulin resistance index, high density lipoprotein cholesterol [HDL-C], and serum uric acid), which have previously been reported to be significantly correlated with the urine pH [4]. The blood urea nitrogen (BUN), as an index of the protein intake (which can affect the urine pH) [16, 17], and other factors that potentially act as proton buffers in the blood (i.e., total protein and Hb) or which contribute to the excretion of protons from the body via urine (i.e., estimated glomerular filtration rate [eGFR]) were also assessed as secondary outcomes. We hypothesized that aerobic exercise at the AT would ameliorate the urine pH levels in elderly women, presumably due to the enhancement of the mitochondrial function in the skeletal muscle.

## Materials and Methods

### The study design and subjects

The current study was secondary analysis of a randomized controlled trial (RCT) [14, 18], which was conducted in order to investigate the efficacy (rather than effectiveness) of aerobic exercise in ameliorating the urine pH; thus a per protocol analysis was performed. From 2004 to 2005, participants were recruited through a public advertisement in a bulletin distributed by a town in Northern Kyushu. Post-menopausal women of ≤85 years of age who were apparently healthy were eligible for inclusion in the present study. The exclusion criteria included any medical conditions that contraindicated exercise, such as cardiovascular disease or orthopedic disease. The eligible participants were assigned to either a step exercise group or a control group using simple, computerized randomization procedures, without stratification or blocking. Basically it was a single-blind trial, and all of the investigators and assessors, with the exception of the assessor of the exercise testing (N.H.), were blinded. With a two-sided alpha value of 0.05, the effect size of urine pH was 0.6 (it was hypothesized that there would be a 10% change from baseline in the exercise group), and the subject number per group was 29, the power calculated was 90%. To investigate the effects of exercise intervention independently of the influence of dietary factors, all of the subjects in the two groups were instructed to not change (and to simply maintain) their normal lifestyle and to continue their usual dietary habits; the participants in exercise group regularly performed step exercises in their home throughout the 12-week study period. The RCT was retrospectively registered in the University Hospital Medical Information Network (UMIN) as “Effect of step exercise on aerobic fitness and progression of atherosclerosis in the elderly” under the registration number UMIN 000026743.

### Exercise testing using a bench step

Submaximal graded incremental exercise tests were conducted using a bench step (height: 15 cm or 20 cm) to assess the AT (which is considered to be a superior index of the aerobic capacity and the intensity of exercise training in home-based training programs), as previously described [19]. The step rhythm was initially set at 40 steps/min, and was increased by 10 steps/min every 4 min, separated by 2-min rest intervals. The heart rate (Polar T31-coded transmitter, Polar Electro, Oy, Kempele, Finland) and the rating of perceived exertion were recorded during the last minute of exercise at each step rhythm. Blood samples (5 μL) were obtained from the earlobe immediately after exercise at each step rhythm, and the blood lactate concentration was measured using a portable blood lactate test meter (Lactate pro, Arkray, Inc., Kyoto, Japan). Oxygen consumption during the bench step exercises was estimated based on the height of the bench step and the step rhythm [19]. Metabolic equivalents (METs) were calculated by comparing the oxygen consumption at rest (3.5 mL/kg/min). The estimated oxygen consumption or METs at the first breakpoint of the blood lactate concentration was judged as the lactate threshold; this exercise intensity defines the AT [20]. The AT data were considered to be missing when the lactate threshold was not assessed due to an insufficient blood sample. The number of subjects in whom the AT was analyzed is shown in the footnotes of the table.

### The home-based bench step exercise training

The exercise regimen was a moderate intensity, home-based aerobic training using a bench step. The participants in the bench step exercise program were instructed to perform the bench step exercises at the AT three times per day (10–20 min per session), with a goal of performing ≥140 min of exercise in their home per week for the 12-week period. In order to adjust the workload, an additional step exercise test was performed at six weeks after the commencement of training, and the revised workload, which corresponded to the newly determined AT was used for the remaining 6 weeks. Exercise sessions were conducted once a week at a community welfare center by an exercise leader in order to maintain the subjects’ motivation. Based on the self-recorded exercise logs that were submitted by a subset of the subjects in the exercise group (*n* = 13), the average length of time spent performing bench step exercises at home was 208 ± 62 min/week over the 12-week period. The participants assigned to the control group were instructed to simply maintain their normal lifestyle and usual dietary habits for the duration of the study.

### The anthropometric indices, blood pressure, and urine and blood sampling, and laboratory assay

The BMI was determined by dividing the body weight (kg) by the square of the height (m^2^). Measurements of blood pressure (systolic blood pressure [SBP] and diastolic blood pressure [DBP]) were obtained in the sitting position after 5 min of rest using an automatic sphygmomanometer (BP-203RV, Nihon Colin, Tokyo, Japan). Before and after the 12-week study period, urine and venous blood samples were obtained from each participant after overnight fasting. The subjects were asked to collect midstream urine from the first discharge in the morning. The urine and separated serum samples were stored until further use at −30 °C. The urine pH was measured once (not in duplicate) using the test paper method (Uropaper-αШ Eiken, Eiken Kagaku, Inc., Tochigi, Japan). This test paper method assesses the urine pH in steps of 0.5 with a range of 5.0 to 9.0. With regard to repeatability, the concordance rate of the measured urine pH levels between the currently used test paper method and a different test paper method (*n* = 90), which was performed according to the manufacturer’s instructions, was 100%. The intake of protein-rich diet may change (or reduce) the urine pH levels [16, 17]; thus, the BUN level, as an index of the protein intake, was measured in the current study according to the urease and leucine dehydrogenase and ammonia avoidance method [21], in order to confirm that the protein intake did not change throughout the study period. The creatinine concentration levels in the serum and urine were measured according to the enzymatic method [22, 23]. The estimated glomerular filtration rate (eGFR), which is an index of the kidney’s function in the production of urine, was calculated using the following formula: eGFR = 194 × serum creatinine value^-1.094^ × age^-0.287^ × 0.739. The glucose, insulin, hemoglobin A1c (HbA1c), TG, HDL-C, serum uric acid, total protein, and Hb levels were measured according to the standard methods, but not in duplicate. The homeostasis model assessment of insulin resistance (HOMA-IR) was calculated as the product of the fasting glucose (mg/dL) and insulin (μU/mL) levels divided by 405 [24]. The HbA1c value was estimated as the National Glycohemoglobin Standardization Program (NGSP) equivalent value, which was calculated as HbA1c (NGSP [%]) = 1.02 × HbA1c (Japan Diabetes Society value [%]) + 0.25% [25].

### Statistical analysis

The main outcome measure was the urine pH. The secondary outcome measures were metabolic parameters (TG, glucose, HOMA-IR, HDL-C, and serum uric acid), which have previously been reported to be significantly correlated with the urine pH [4]. The BUN as an index of the influence of the protein intake on the urine pH levels and other factors that potentially act as proton buffers in the blood (i.e., total protein, Hb) or which contribute to the excretion of protons from the body via urine (i.e., eGFR), were also assessed as secondary outcomes. As noted above, a per protocol analysis was performed. The values are shown as the mean ± SE. Intra-group comparisons of the data obtained before (baseline) and after the 12-week intervention were performed using Wilcoxon’s signed-rank test. Comparisons of the inter-group differences (between the exercise and control groups) in the baseline data were made using the Mann-Whitney *U* test. To identify the clinical variables that potentially explain the exercise-induced changes in the urine pH, the significance of correlations between the changes in the significantly changed variables and the change in the urine pH was assessed in the exercise group according to the Spearman’s rank correlation coefficient. As for the primary outcome of the urine pH, the post-intervention values adjusted for the pre-intervention values of each group, as well as the difference between the adjusted post-intervention values of the two groups (i.e., the estimated effect size) plus its precision (i.e., 95% confidence intervals) were analyzed using an analysis of covariance. All of the statistical analyses were performed using the SAS software program (version 9.3 for Windows, SAS Institute, Cary, NC, USA). A *p* value of <0.05 was considered to indicate statistical significance.

## Results

### The effects of the bench step exercise on the anthropometric indices, aerobic capacity, blood biochemical parameters, and urine creatinine levels

**Fig. 1:**
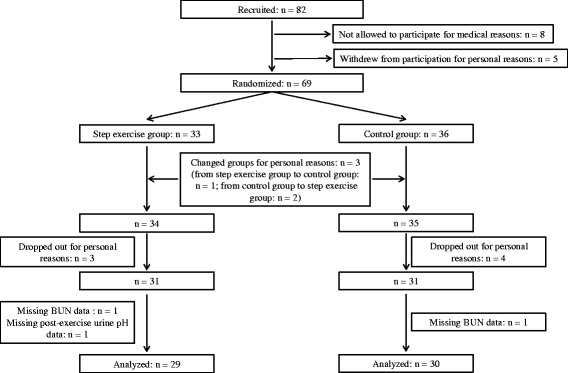
A flow diagram of the study participant. Sixty-nine participants were randomly assigned to the bench step exercise group or the control group. A total of 59 participants (step exercise group, *n* = 29; control group, *n* = 30) remained for the analysis. *BUN*, blood urea nitrogen

Figure 1 shows a participant flow diagram. After the 69 participants were randomly assigned to the step exercise or control groups, one participant who was originally assigned to the exercise group changed to the control group while two participants who were originally assigned to the control group changed to the exercise group based on the subjects’ preference. Among the 69 remaining participants (exercise group, *n* = 34; control group, *n* = 35) seven participants (exercise group, *n* = 3; control group, *n* = 4) dropped out for personal reasons. Two more participants with missing BUN data (exercise group, *n* = 1; control group, *n* = 1) and one participant from the exercise group whose post-exercise urine pH data were missing were excluded from the analysis. Consequently, 59 subjects (exercise group, *n* = 29; control group, *n* = 30) were included in the per protocol analysis. In the exercise group, the baseline body weight and BMI were higher, while the serum uric acid level and eGFR were lower in comparison to the control group (Table 1). The baseline urine pH levels of the two groups were similar. In the exercise group, the BMI decreased, and aerobic capacity increased (as indicated by AT) with the exercise program, as previously reported [14]. The serum uric acid showed statistically significant improvement after the exercise program; the changes in these variables (BMI, AT, serum uric acid) were not observed in the control group. The other metabolic parameters (glucose, HOMA-IR, TG, HDL-C) were not significantly affected by the exercise. As for the indices related to the blood buffering capacity and the urine production function of the kidney, there were no changes in two buffers in circulation (total protein and Hb) or the index of kidney function (eGFR values) in either the exercise or control groups. The BUN value and the BUN/creatinine ratio, as indices of the dietary protein intake, were also not changed after the 12-week study in either of the groups. Additionally, the urine creatinine levels, as an index of the degree of urine concentration, was unchanged during the study period in both groups (exercise group: before 103 ± 9, after 110 ± 10 mg/dL, *p* = 0.34; control group: before 110 ± 13, after 99 ± 12 mg/dL, *p* = 0.46).

**Table 1:** The characteristics of the study subjects at baseline

	Step exercise group (*n* = 29)	Control gourp (*n* = 30)	*p* value^a^
Age (years)	70.2 ± 1.1	70.2 ± 1.1	0.82
Height (cm)	148.7 ± 1.0	149.0 ± 0.9	0.83
Weight (kg)	53.9 ± 1.4	49.8 ± 1.0	<0.05
BMI (kg/m^2^)	24.4 ± 0.7	22.5 ± 0.5	<0.05
AT (METs)^b^	4.2 ± 0.2	4.3 ± 0.2	0.79
SBP (mmHg)	138 ± 4	140 ± 3	0.67
DBP (mmHg)	77 ± 2	76 ± 2	0.75
Glucose (mg/dL)	100 ± 6	95 ± 2	0.86
Insulin (μU/mL)	5.8 ± 0.5	5.0 ± 0.4	0.18
HOMA-IR	1.4 ± 0.1	1.2 ± 0.1	0.13
HbA1c (%)	5.90 ± 0.23	5.59 ± 0.10	0.70
TG (mg/dL)	103 ± 9	100 ± 8	0.88
HDL-C (mg/dL)	65 ± 3	69 ± 3	0.67
Serum uric acid (mg/dL)	4.4 ± 0.2	5.1 ± 0.2	<0.01
Total protein (g/dL)	7.6 ± 0.1	7.6 ± 0.1	0.87
Hb (g/dL)	13.3 ± 0.2	12.9 ± 0.2	0.12
BUN (mg/dL)	15.5 ± 0.7	15.3 ± 0.7	0.83
BUN/creatinine ratio	23.6 ± 1.2	26.4 ± 1.5	0.25
eGFR (mL/min/1.73 m^2^)	67.6 ± 2.4	78.1 ± 3.5	<0.05
Urine pH	6.1 ± 0.1	5.9 ± 0.1	0.27

Values are the mean ± SE

*AT* anaerobic threshold, *BMI* body mass index, *BUN* blood urea nitrogen, *DBP* diastolic blood pressure, *eGFR* estimated glemerular filtration rate, *Hb* hemoglobin, *HbA1c* hemoglobin A1c, *HDL-C* high density lipoprotein cholesterol, *HOMA-IR* homeostasis model assessment of insulin resistance, *LDL-C* low density lipoprotein cholesterol, *MET* metabolic equivalent, *SBP* systolic blood pressure, *TG* triglyceride

^a^The *p* value for the inter-group comparison of baseline data between the exercise group and the control group

^b^Based on 26 subjects in the control group

### The effects of the moderate step exercise on the urine pH levels

**Fig. 2:**
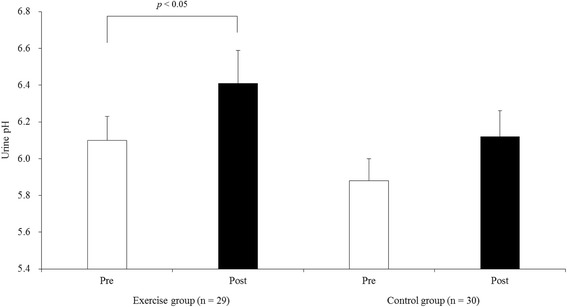
The changes in the urine pH after the 12-week study in the exercise group and the control group. The columns represent the mean ± SE. The intra-group comparisons were made using Wilcoxon’s signed-rank test

The intra-group comparison analysis using Wilcoxon’s signed-rank test showed that the urine pH levels were significantly increased in the exercise group (pre-intervention 6.10 ± 0.13, post-intervention 6.41 ± 0.18, *p* < 0.05), whereas the urine pH of the control group did not change to a statistically significant extent (pre-intervention 5.88 ± 0.12, post-intervention 6.12 ± 0.14, *p* = 0.15) (Fig. 2). The post-intervention values adjusted for the pre-intervention values (in each group), and the difference between the adjusted post-intervention values of the two groups (i.e., the estimated effect size) plus its precision (i.e., 95% confidence intervals) are shown in Table 2. The estimated effect size (0.15) was small and its 95% confidence interval straddled 0. Similarly, the adjusted mean urine pH change (before and after the intervention) in the exercise (0.35 [95% confidence interval 0.06 − 0.63]) and control (0.20 [-0.08 − 0.48]) groups did not differ to a statistically significant extent (*p* = 0.46).

**Table 2:** The adjusted post-intervention values, estimated effect size, and precision regarding the effect of step exercise training on the urine pH

	Post-intervention values^a^	95% confidence interval	Effect size^a^	95% confidence interval
Exercise group	6.34	6.05–6.62	0.15	−0.25–0.55
Control group	6.19	5.91–6.47		

^a^Adjusted for pre-intervention urine pH values

We did not observe any significant correlation between the change in the urine pH after the exercise and the changes in BMI (*ρ* = 0.26, *p* = 0.17) and the serum uric acid level (*ρ* = −0.12, *p* = 0.53). Another factor that showed significant changes after the exercise program was the AT; however, the change in this index of aerobic capacity was not significantly correlated with the change in the urine pH after the exercise program (*ρ* = −0.28, *p* = 0.14).

## Discussion

To our knowledge, the current study is the first to investigate whether or not aerobic exercise improves the urine pH in humans. Despite the crucial importance of metabolic acidosis in the development of insulin resistance and other chronic diseases [6, 8, 9], there is only one report on an effective approach to improve metabolic acidification, in which a diet containing propolis (a natural compound derived from honeybee product) was found to elevate the pH of interstitial fluids and simultaneously enhance insulin sensitivity in a rodent model of type 2 diabetes mellitus [26].

In the present study, the pre-post comparison of the urine pH data using Wilcoxon’s signed-rank test showed a significant increase in the urine pH levels of the exercise group, while there was no significant change in the urine pH levels of the control group. However, when the degree of the effect of exercise was assessed based on the estimated effect size and its precision (95% confidence interval), the effect size was small (0.15) and the confidence interval straddled 0. Thus, the magnitude of the effect of exercise training on the urine pH could not be considered significant. This negative result is attributed to both the small degree of increase in the average urine pH in the exercise group (5.1%) and the unexpected nonsignificant increase in the urine pH of the control group (4.1%; Wilcoxon’s signed-rank test, *p* = 0.15). We were not able to clearly explain why the urine pH levels of the control group tended to increase.

Previous reports showing that either a higher BMI value or an increase in the uric acid level was linked with more acidic urine [2, 3, 27]. The participants in our exercise group showed significant changes in these two parameters and AT after the exercise program. However, we did not observe any significant association between the change in the urine pH after the exercise and the changes in BMI, serum uric acid level, or AT. Additionally, the indices of the blood buffers (total protein and Hb) as well as the index of kidney function (eGFR) were not altered by the exercise. Thus, in the current study, we were unable to identify the clinical factors that might contribute to the elevation of the urine pH level after the exercise. Further studies will be needed to identify the factors that determine the exercise-induced amelioration of the urine pH level.

In the recent reviews on the body pH status and metabolic diseases [8, 9], the acidification of body fluids (especially interstitial fluid in skeletal muscle) is considered to be one of the important mechanisms underlying insulin resistance and type 2 diabetes. Thus, we hypothesized that the acidification of the body fluids, including urine, is a cause (rather than a result) of insulin resistance and that the amelioration of the urine pH status with exercise may precede the exercise-induced improvement in insulin resistance. The 12-week duration of exercise training at the AT was sufficient for enhancing insulin sensitivity in younger subjects (24.8 ± 1.8 years of age) [28]; however, the duration (12 weeks) of the current program might have been too short to achieve the amelioration of the urine pH and insulin sensitization in the elderly subjects.

The current study is associated with several limitations. The usage of 24-h accumulated urine is ideal for precisely assessing the whole-day pH status; however, in the current study the urine sample was only collected once in the morning, as it is a highly practical method of reliably assessing the metabolic acidosis status [4]. Since the present subjects were not provided with the same fixed diet during the study period, we cannot totally exclude the possibility that their diet (in particular, protein-rich food which can induce urine acidification) might have influenced the urine pH status; however, we asked each subject not to change and to simply maintain their usual dietary habits throughout the study period and we confirmed that the BUN concentration levels (as an index of protein intake) were not altered during the current study.

## Conclusion

The current study was a secondary analysis of an RCT that was performed to investigate the effect of aerobic exercise on the urine pH levels in older women. Although the pre-post comparison analysis using Wilcoxon’s signed-rank test showed that the urine pH levels were only significantly increased in the exercise group, the degree of the effect was considered to be insignificant based on the estimated effect size and its precision. Thus, we could not clearly conclude that aerobic exercise training has a beneficial effect on the urine pH. Further well-designed RCTs should be conducted to determine whether aerobic exercise is truly able to ameliorate urine acidification.
